# Acute Bacterial Sinusitis With Epidural and Subdural Involvement

**DOI:** 10.7759/cureus.34849

**Published:** 2023-02-10

**Authors:** Chelsea-Jane E Arcalas, Daniel A Reich, Samari A Blair, Nicole M Paradise Black

**Affiliations:** 1 College of Medicine, University of Florida, Gainesville, USA; 2 Department of Pediatrics, University of Florida, Gainesville, USA

**Keywords:** intracranial epidural abscess, pediatric headache, rhinosinusitis, intracerebral abscess, subdural empyema

## Abstract

Sinusitis is a common childhood infection with potential for rare intracranial complications. These neurologic sequelae can lead to serious morbidity and mortality if not addressed promptly. We describe a case of suspected sinusitis in a 13-year-old male complicated by a superior sagittal sinus thrombosis along with subdural and epidural empyemas.

## Introduction

Pediatric sinusitis is generally considered a mild infection and is commonly found in the setting of viral or bacterial etiologies [1]. Classic symptoms of congestion, nasal obstruction, and facial pressure overlap with those of viral upper respiratory infections, making diagnosis a challenge. Symptoms usually resolve with conservative management in the setting of a viral etiology; however, oral antibiotics are warranted when the condition worsens [1]. Although rare, extracranial and intracranial complications may occur, with orbital complications including orbital cellulitis and abscess formation being some of the most frequently reported.

Intracranial complications including subdural empyema, epidural abscess, meningitis, or cerebritis are particularly important to recognize due to the possible rapid clinical deterioration and significant morbidity and mortality associated with this condition [2]. Similar intracranial complications may also present in the setting of otitis media, another highly prevalent infection in childhood [3]. Common intracranial complications of acute otitis media are otitic hydrocephalus, extradural abscess, and lateral sinus thrombosis. Those of chronic otitis media include the aforementioned as well as meningitis, cerebral abscess, and encephalitis [4]. It is noted in the literature that intracranial complications may account for 30% of all complications of otitis media.

Aside from these rare conditions, acute mastoiditis is consistently reported as the most frequent complication of otitis media in the pediatric population, with a majority of cases occurring at around ages two to three years old [5]. Predictably, associated intracranial complications of acute mastoiditis are similar to those of sinusitis and otitis media. In one retrospective review, the top two conditions of this category were reported to be sigmoid sinus thrombosis and intracranial abscess. Subperiosteal abscess was reported as the most common complication overall [6]. 

This case report describes an older pediatric patient who developed a superior sagittal thrombosis, subdural empyema, and epidural empyema after a suspected sinus infection. We describe his clinical course as well as his unique neurologic recovery.

## Case presentation

A 13-year-old male with no significant past medical history presented to the pediatric emergency department (ED) with a five-day history of a right-sided frontal headache, decreased appetite, intermittent subjective fevers, and right orbital swelling. Per the child’s mother, the patient never complained of headaches before the presentation and attributed them to the cessation of routine energy drink consumption. The patient subsequently developed intermittent fevers and was treated supportively at home with acetaminophen. He had an associated decrease in appetite but was tolerating clear liquids. The mother also noticed decreased activity, as the child did not seem interested in playing sports as much as usual. On the morning of the presentation, the patient had transient numbness of the left upper and lower extremities that lasted approximately five minutes before spontaneously resolving. Approximately 10 minutes later, the patient had another episode of numbness in the same distribution, which also resolved spontaneously. These two transient episodes of numbness along with progressive right eye swelling prompted the mother to bring the patient to the ED. 

**Figure 1:**
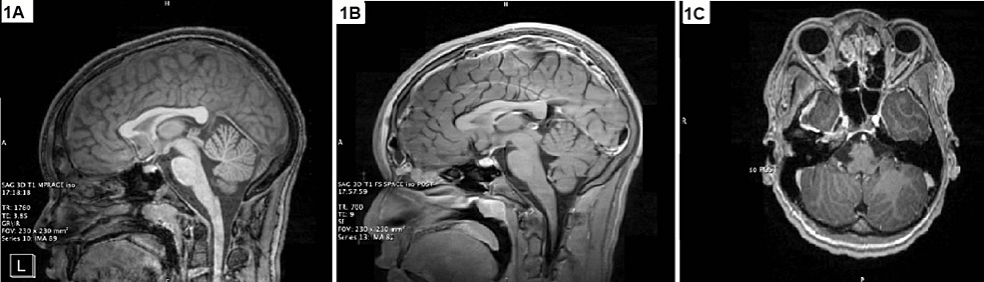
(A-C) Initial brain magnetic resonance imaging with early formation of a right frontal, maxillary, and ethmoid sinusitis and concerns for superior sagittal sinus thrombosis, small subdural empyema, and mild right frontal leptomeningeal enhancement

In the ED, the patient was febrile to 39.1°C and received acetaminophen. The patient was started on maintenance intravenous fluids and empiric antibiotics consisting of clindamycin and piperacillin-tazobactam. Initial laboratory workup showed the following elevated lab values: a C-reactive protein of 225 (normal <10 mg/L), an international normalized ratio of 2.1 (normal </= 1.1), a prothrombin time of 24 (normal 11-13.5 seconds), and an erythrocyte sedimentation rate of 43 (normal 0-22 mm/hr). There was no leukocytosis. Because of presenting symptoms of headache and numbness, a computed tomography (CT) angiography scan of the head and neck with and without contrast was obtained promptly. The study was notable for a chronic appearing, multifocal, nonocclusive thrombus in the superior sagittal sinus. It also showed moderate paranasal sinus mucosal changes with pneumatized secretions in the right maxillary sinus and posterior ethmoid air cells as well as chronic osteitis changes in the right maxillary sinus walls. Because of these sinus findings, radiology suggested that these were secondary to acute on chronic sinusitis. Pediatric neurology recommended further imaging given the concerns for thrombosis and meningitis. Initial magnetic resonance imaging (MRI) revealed mild right frontal leptomeningeal enhancement, subtotal opacification of the frontal sinuses and right maxillary sinus with air-fluid levels, and severe mucosal inflammatory changes of the anterior ethmoid air cells bilaterally. There were also concerns for a small right frontal subdural empyema and subocclusive filling defects in the superior sagittal sinus. These MRI findings are demonstrated in Figures 1A-1C. Lumbar puncture was obtained due to concerns for meningitis, revealing the following: glucose of 74 (normal 50-80 mg/100mL), protein of 27 (normal 15-60mg/dL), white blood cells of 4 (normal 0-5 cells/µL), and red blood cells of 12 (20-40 mg/dL). The patient was admitted to the pediatric hospital medicine service for continued antibiotics and further management. 

The following morning, the patient had acute neurologic changes, including left cranial nerve VI and VII palsies as well as 2/5 left upper extremity grip weakness. Several consults were placed including neurosurgery (NSGY), infectious disease (ID), ophthalmology, otolaryngology (ENT), and hematology/oncology. He was subsequently transferred to the pediatric intensive care unit (PICU) given the acute neurological changes and the need for frequent neurological exams. NSGY did not recommend surgical intervention at this time due to the small size of the empyema. ENT recommended sinus aspiration for culture and possible drainage. The hematology and oncology team recommended anticoagulation following the sinus aspiration/drainage. In the PICU, the patient was transitioned to vancomycin, ceftriaxone, and metronidazole per ID recommendations. A repeat CT showed progression of the subdural empyema with an increase in soft tissue swelling. 

On hospital day three, ENT performed a functional endoscopic sinus surgery, which included a bilateral total ethmoidectomy and sphenoidectomy, frontal sinusotomy, and maxillary antrostomy. On hospital day five, NSGY performed a right frontal subdural empyema evacuation. The patient was started on Lovenox treatment for superior sagittal sinus thrombosis with a therapeutic goal of anti-Xa levels of 0.5-1 units/mL. Cultures drawn during the NSGY evacuation grew *Klebsiella spp.* from the sinuses and *Gamella moribilum* from the empyema. ID switched his antibiotic regimen from vancomycin to linezolid and continued metronidazole and ceftriaxone. Inflammatory markers gradually improved, as did a leukocytosis that developed during the hospitalization. The patient was transferred from the PICU to the general inpatient service on hospital day five for further management. 

**Figure 2:**
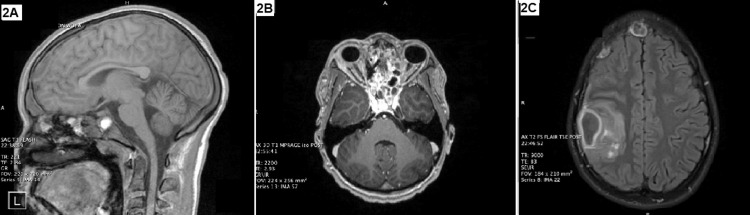
(A-C) Brain magnetic resonance imaging on hospital day 14 revealed a collection overlying the right frontotemporal lobe that increased in size, new surrounding vasogenic edema, and parenchymal enhancement.

**Figure 3:**
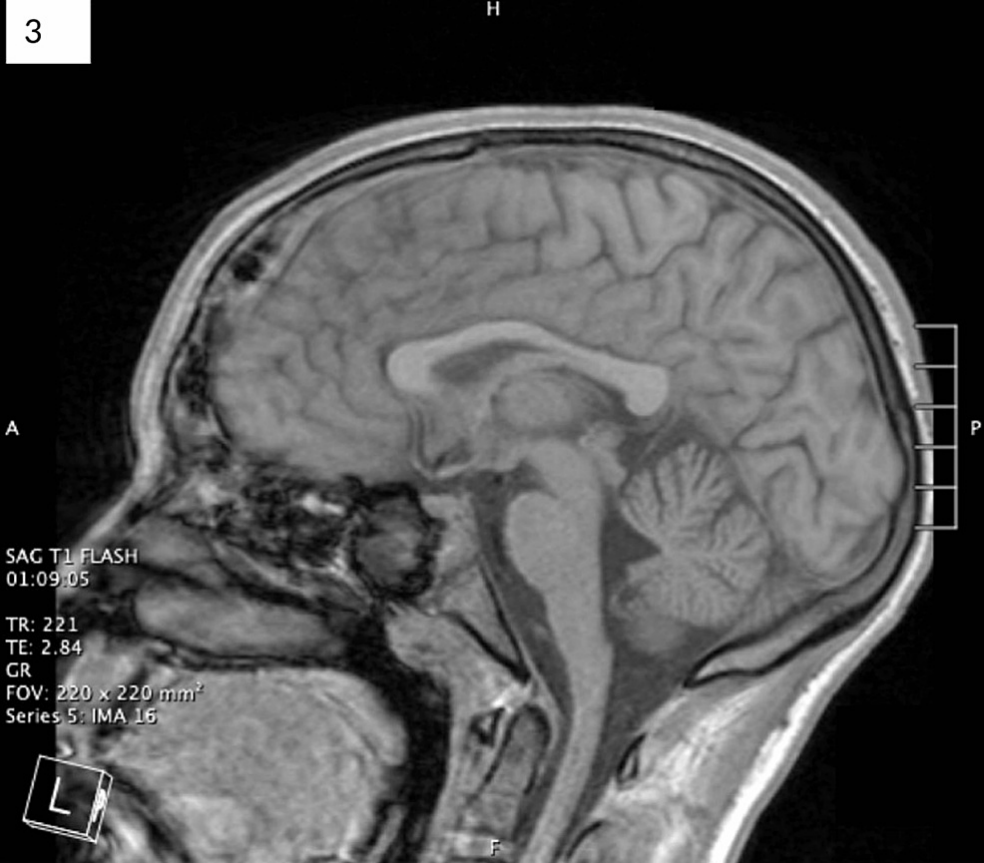
Final brain magnetic resonance imaging on hospital day 21 before the patient was discharged home. Imaging revealed continued improvement in the areas of involvement from the midline over the high frontal convexity and multiloculated over the right frontal lobe.

Repeat brain MRI on hospital day 14 showed an increasing size of the right subdural empyema and a new midline frontal epidural empyema, as well as new surrounding vasogenic edema and parenchymal enhancement (Figures 2A-2C). Based on this imaging, he underwent needle aspiration of the two intracranial empyemas. Following surgery, the patient continued to show clinical improvement with full recovery of the left cranial nerve VI and VII palsies. He started working with physical and occupational therapy three times a week to regain strength. A repeat brain MRI on hospital day 21 showed near complete resolution of the intracerebral abscess with residual vasogenic edema (Figure 3). He was discharged home with a peripherally inserted central catheter for long-term antibiotic therapy with ceftriaxone and metronidazole and subsequent outpatient follow-up with ID, ENT, and NSGY. 

## Discussion

Previous literature of case reports/series demonstrates that most patients with neurologic involvement secondary to complicated sinusitis show eventual resolution of neurological deficits [7]. However, patients with certain complications, including subdural empyemas and dural sinus thromboses, often suffer from significant morbidity [8-10]. This includes residual motor neurological deficits, cognitive and behavioral problems, or persistent seizures, potentially affecting up to 50% of survivors [10]. Mortality and long-term neurological deficits still occur frequently despite advancements in workup and treatment [8].

In a case series published by the Journal of the American Medical Association in 2006, researchers at the Children’s Hospital of Philadelphia followed 25 children with intracranial complications secondary to sinusitis who were treated between January 1, 1999, and April 30, 2004 [7]. Most of the patients in this series were male (76%, n=19), with a mean age of 13.5 years. All patients were healthy at presentation, with a median duration of 12 days of symptoms including headache, fever, nausea, and/or vomiting. Fifty-eight percent of patients had some form of neurological deficit at the time of presentation, with altered mental status being the most common. The presence of neurologic signs and symptoms at the time of presentation was correlated with the type of sinusitis complication as well as prognosis. In the patients with no central neurologic findings, nearly all patients had an epidural abscess. In contrast, patients presenting with at least one neurologic finding were more likely to have a more serious complication including meningitis, encephalitis, cerebral abscess, subdural empyema, and dural sinus thrombosis. In addition, patients in this group were more likely to have short-term sequelae including seizures and hemiparesis. Eight patients had to be placed on prophylactic phenytoin of various duration; six of these patients were identified as having subdural empyema. Epidural abscesses have a more favorable prognosis because they progress more slowly in comparison to subdural empyema. The subdural empyema spreads rapidly and freely within a preformed space, typically resulting in a more fulminant, acute presentation with earlier development of neurologic deficits. 

In a more recent retrospective study also conducted at the Children’s Hospital of Philadelphia in 2021 [8], researchers looked at 54 patients admitted for operative management of sinusitis with intracranial extension. Like the older case series, there was a male predominance among the patients, and the average age was 11 years. The median duration of symptoms before hospitalization was seven days and less than one-third of patients reported neurological symptoms at the time of presentation. It is important to note that in this study, nearly all (89%) of patients had a healthcare visit in the days leading up to admission, and 50% were prescribed antibiotics. Those who had a subdural empyema proved to have a poorer prognosis and/or further complications; those who required repeat surgery were significantly more likely to have been diagnosed with a subdural empyema. 

The uniqueness of this case arises from the multiple self-resolving episodes of focal neurologic deficits and, ultimately, full neurologic recovery. There is limited information detailing the progression and spontaneous resolution of simultaneous neurological deficits, cranial nerve palsies and hemiparesis, after having multiple areas of involvement. There are no standard guidelines or recommendations for the management of intracranial complications of sinusitis; treatment varies by the clinical institution. While most pediatric patients with intracranial complications underwent some form of neurosurgical and otolaryngological intervention, gold-standard surgical management has not been fully established [9]. 

## Conclusions

The current case and prior literature emphasize the importance of providers having a high index of suspicion for complications in patients with known sinusitis who present with symptoms greater than one week. This is especially true for those who do not improve despite appropriate antibiotic treatment. Providers should have a low threshold for obtaining prompt neuroimaging with CT or MRI, as symptoms of infection extension may be subtle. Simultaneously, broad-spectrum intravenous antibiotics should be initiated after prompt recognition. This case demonstrates that care of these patients should be multidisciplinary, with a team of hospitalists, intensivists, neurologists, neurosurgeons, otolaryngologists, infectious disease specialists, and hematologists in the event of thrombosis, as well as rehabilitation (physical, occupational, and speech therapists) for those with residual neurological deficits. We hope this case report adds to the literature of intracranial complications following pediatric sinusitis and provides further guidance for subsequent management of these complications.

## References

[1] Ramadan HH, Chaiban R, Makary C (2022). Pediatric rhinosinusitis. Pediatr Clin North Am.

[2] Muzumdar D, Biyani N, Deopujari C (2018). Subdural empyema in children. Childs Nerv Syst.

[3] Poutoglidis A, Tsetsos N, Keramari S, Skoumpas I, Vlachtsis K, Kilmpasanis A, Fyrmpas G (2021). Sigmoid sinus thrombosis as a complication of acute Otitis Media in a 6-year-old male. Ear Nose Throat J.

[4] Penido Nde O, Chandrasekhar SS, Borin A, Maranhão AS, Gurgel Testa JR (2016). Complications of otitis media - a potentially lethal problem still present. Braz J Otorhinolaryngol.

[5] Loh R, Phua M, Shaw CL (2018). Management of paediatric acute mastoiditis: systematic review. J Laryngol Otol.

[6] Favre N, Patel VA, Carr MM (2021). Complications in pediatric acute mastoiditis: HCUP KID analysis. Otolaryngol Head Neck Surg.

[7] Germiller JA, Monin DL, Sparano AM, Tom LW (2006). Intracranial complications of sinusitis in children and adolescents and their outcomes. Arch Otolaryngol Head Neck Surg.

[8] Otto WR, Paden WZ, Connors M (2021). Suppurative intracranial complications of pediatric sinusitis: a single-center experience. J Pediatric Infect Dis Soc.

[9] Patel NA, Garber D, Hu S, Kamat A (2016). Systematic review and case report: intracranial complications of pediatric sinusitis. Int J Pediatr Otorhinolaryngol.

[10] Bruner DI, Littlejohn L, Pritchard A (2012). Subdural empyema presenting with seizure, confusion, and focal weakness. West J Emerg Med.

